# Gaining Longitudinal Accounts of Carers' Experiences Using IPA and Photograph Elicitation

**DOI:** 10.3389/fpsyg.2020.521382

**Published:** 2020-12-04

**Authors:** Val Morrison, Karina Williams

**Affiliations:** School of Psychology, Bangor University, Bangor, United Kingdom

**Keywords:** caregivers outcomes, willingness to care, caregiver accounts, qualitative methods, motivations to care, longitudinal

## Abstract

Fluctuations in positive and negative caregiving experiences remain only partially explained as the significant variability over time of potential predictive factors themselves is understudied. The current study aims to gain considerable insight into caregiving experiences and perceptions over time by using photovoice methodology to support semi-structured interviews. A case study, longitudinal design is taken with three female caregivers who provide detailed insight into their caregivers' experiences over a 12 month period. The interview transcripts were analyzed using IPA- Interpretative Phenomenological Analysis. This innovative combination of methods resulted in the emergence of three related themes which included consuming the role, feeling consumed by the role, and letting go of the role. The idiographic approach taken allowed both within case differences to be examined over time, and also between carer differences to be highlighted. Implications of illness type and its characteristics, and of attachment and relationship quality with the care recipient were seen in terms of how and when the caregivers moved between the themes identified. The use of others' support or respite care is examined vis-a vis caregiver's own beliefs, emotions, relationship attachment and motivations to care. Caregivers self-efficacy beliefs also shifted over time and were influential in caregiver experience as the care recipient condition or needs changed. No previous studies have found that negative caregiving consequences are, in part, under volitional control and yet our data on the underlying reasons for consuming caregiving or allowing themselves to consume, would suggest this may in part be true. This is important because it suggests that interventions to support caregivers should address relational and motivational factors more fully.

## Introduction

Caregiving experience embraces both positive and negative experiences, (e.g., Rohr and Lang, 2014; Roth et al., 2015). Carers' experiences of positive (e.g., satisfaction, fulfillment, purpose, and carer-recipient cohesiveness) or negative (strain, depression, and anxiety) caregiving outcomes can change over time (Silverberg-Koerner and Baete-Kenyon, 2007). Fluctuations in positive and negative caregiving experiences remain only partially explained as the significant variability of potential predictive factors themselves is understudied (e.g., Pihet et al., 2017; Van Knippenberg et al., 2017; van Knippenberg et al., 2018). The negative consequences of caregiving have been found to have a detrimental impact on carers' psychological and physical well-being (Parveen et al., 2011; Williams et al., 2014; Angelo and Egan, 2015; Faucher and Garner, 2015; Liu et al., 2019) and can continue even after the role has been relinquished (Seddon et al., 2002; Boerner et al., 2004).

Current carers, who advocate determination to provide care, report motivations to adopting and maintaining caregiving out of love, affection, guilt, obligation, and protection (Ribeiro and Paul, 2008; Williams et al., 2014), highlight a range of different emotional states. There is however a limited body of work addressing caregiving motivations and how they may change over time. The relationship between the caregiver and recipient, and intrinsic motivations to care (e.g., principles, love, caring nature) as opposed to extrinsic motivations (e.g., out of guilt or expectation) are important to caregiver well-being (Lyonette and Yardley, 2003; Sorensen et al., 2008; Williams et al., 2014). Few qualitative studies have however followed carers' experiences prospectively, once the caregiving role is adopted, thus failing to capture whether willingness and motivations to care are dynamic in nature and whether they are related to the experience of gains and losses. Interpretative techniques that can also reveal the beliefs and emotions underpinning such motivations may prove particularly fruitful when applied longitudinally. Thus, our research aims to explore current caregiving and willingness to continue to provide care using several longitudinal caregiver case studies and using Interpretative Phenomenological Analysis (IPA). Our research will also explore whether factors such as illness type, perceptions of stress, coping and self-efficacy determine perceived gains, caregiving ability and willingness to care in the future.

Stress and coping theorists consider that the individual response to circumstances differs between persons, contexts and over time and is dependent upon factors such as the timing and type of the stressful event. The transactional stress model (Lazarus and Folkman, 1984) is one such theory which proposes that individuals experience events as positive or negative (eustress or stress/distress) depending on their appraisals of the stressor and its characteristics and context. Physical illnesses can place many family members under overwhelming stress. In our aging society, living with chronic disease and disability is becoming increasingly commonplace and it is family members who are typically expected to provide physically and emotionally demanding care for relatives. The caregiving role and the demands it places on the individual carer is likely to be affected by the nature of the care recipient's condition. For example, using just two conditions as an example, the onset of a stroke is typically sudden and unexpected by the stroke patient and their family, whereas the onset of a dementia and its' related symptoms is gradual. In the case of stroke, a person is typically faced with an acute loss of motor or language skills with family members having to meet immediate changes to their lives that can last for many months or longer. In contrast, because the onset and progression of dementia symptoms is often gradual, carers face incremental changes in their loved one's behavior and cognitive functioning often long before a diagnosis is given, and some adjustments within a relationship may already have emerged. Research has found that executive functioning and personality deteriorates quicker in those diagnosed with frontotemporal dementia (FTD) in comparison to those diagnosed with Alzheimer's Disease (AD). Informal carers of people diagnosed with FTD (usually a relative, sibling or close friend) may have to deal with more behavioral, cognitive, and personality changes in the care recipient than is the case for other dementias or degenerative illnesses (Rosness et al., 2008; Nicolaou et al., 2010). Charmaz (2014) presented a theoretically informed typology of illness experiences following qualitative research taking a grounded theory approach to how illness is experienced in relation to time. From this perspective stroke and dementia represent two different types of illness and thus in the current study we compare the experiences of three carers of a loved one who has had either a stroke or have been diagnosed with dementia.

We draw from Interpretative Phenomenological Analysis (IPA), an idiographic technique adapted from phenomenological analysis and hermeneutics to gain insight into the individual experience (Smith et al., 2009). This method is well-suited to addressing questions of individual caregiving motivations and how carers make sense of their role as their personal experiences fluctuate. Most IPA caregiving research has employed a cross-sectional design (Hunt and Smith, 2004; Bolas et al., 2007; Quinn et al., 2008; Hill et al., 2009; Dickson et al., 2010), although Smith (Smith, 1999; Smith et al., 2009) used IPA longitudinally to study four women's experience of becoming a mother from before to after giving birth. Analyses at both the individual case level and between-case comparisons provided in-depth accounts of women's transitional experiences. The authors assert a smaller sample size enables greater transparency of lived experience over time (Smith et al., 2009, p. 51-52). To our knowledge caregiving experience over time in the context of chronic illness has not been examined using IPA, although cross-sectional evidence has highlighted mainly negative consequences of caregiving such as: loss of personal identity; uncertainty; deteriorated carer-recipient relationship quality; and experience of distress. Notably however, the majority of carers included in these studies were at the start of their caregiving role, yet the balance between positive and negative experiences is likely to vary depending on the length of carer experience (e.g., Rohr and Lang, 2014).

We conduct IPA on a small sample, longitudinal design with three case studies in order to gain detailed insight into caregivers' experiences over time. To facilitate capturing caregiving experiences our design invited caregivers to take photographs of their experiences in order to stimulate subsequent in-depth discussion. The technique of photograph elicitation interviews is relatively new to health psychology research and invites the participant to take photographs expressing their individual experiences of a situation. For example, Aubeeluck and Buchanan (2006) captured the experience of Huntington's disease from the perspective of five carers who took photographs signifying their loneliness, lack of time, lack of support and a sense of loss. Williams et al. (2014) employed the method with caregivers across a range of chronic health conditions to develop understanding of the meanings attached to caregiving.

The current study aims to gain considerable insight into caregiving experiences and perceptions over time by combining photovoice methodology with phenomenological analysis, specifically IPA. This has been done before but with differing integration of methods, topics, or analytical method. Papaloukas et al. (2017) gained a holistic understanding of lesbian, gay, bisexual and trans (LGBT) people living with MS and men diagnosed with breast cancer using such methods in combination and in their analysis focused on the analysis of photographs with only minimal summary of emergent themes. Faucher and Garner (2015) used focus group discussions combined with photovoice methodology amongst female family caregivers and analyzed experiences by comparing themes which emerged from the photovoice reflective/description sheets completed by participants when taking their photos with a thematic content analysis of transcripts generated in the cross-sectional focus groups. Given the personal and dynamic nature of family caregiving, longitudinal, one-on-one interviews could possibly have captured even more intricate detail than this cross-sectional focus group data afforded. Using photovoice methodology and adopting a “*generic qualitative analysis technique*” Angelo and Egan (2015) explored experiences of caregivers of ill family members who had less than a year to live. They found that the combination of stories/narratives with reflection on visual data based on personal lived experiences enlightened understanding of the sensitive and often stressful situation caregivers faced. However, despite caregivers in this study meeting with the interviewer multiple times this paper did not report on analyzed experiences over time.

To date, to our knowledge, no published qualitative research has explored caregiving experiences through combining methodology of photovoice elicitation interviews *with* IPA analysis using a longitudinal case study approach. Longitudinal case studies enable greater insight into experiences by providing greater contextual understanding into the lifeworld experience of the caregiver (Osborn and Smith, 1998; Vasileiou et al., 2018) by substantiating the chronology of caregiving experiences and perceptions and any changes over time (e.g., in terms of impact on caregivers willingness to care in the future). Given the above, our research therefore sought to gain in-depth longitudinal accounts of carer experiences using a case study approach with photograph elicitation interviews and applying a phenomenological analysis (IPA).

## Method

### Participants

Caregivers who had taken part in a longitudinal quantitative study (*N* = 78, Williams, unpublished PhD manuscript) were additionally invited to take part in a qualitative photo-elicitation interview study. Thirteen carers expressed interest in participating in this study and took part in the first interview (Williams et al., 2014). Of these, 12 were family members, one was a close friend; 8 were providing care for a loved one with dementia, 5 for a loved one who had suffered a stroke. All but one were female and their ages ranged from 33 to 73 years. Three female Caucasian carers were purposively selected for longitudinal in-depth case study analysis on the basis of their living with the care recipient and providing the main source of full time care to the care recipient but who could also complete the interviews not in the presence of the care recipient. These carers completed three interviews over a period of 12 months. These carers had been providing care for more than 2 years. We have assigned pseudonyms to carers: Dawn aged 33, cared for her husband aged 54 for 3 years who had been diagnosed with FTD; Betty aged 57, cared for her mother aged 90, for 3 years who was a recovering stroke patient; and Susan, aged 64, supported her mother, aged 95, for 5 years who was diagnosed with dementia. Betty and Susan were retired, and Dawn worked part time.

This sample size of three case studies captured longitudinally over three time points (i.e., nine interviews) is considered methodologically appropriate and a sufficient number of interviews for conducting IPA in a robust manner (Smith et al., 2009, p. 51-52).

### Design

In this qualitative longitudinal case study, we conducted semi-structured interviews with three family carers at three time points: baseline (more than 2 years caregiving), six and 12 months from baseline.

### Procedure

This study received the appropriate institutional ethical and research governance approval from Bangor University School of Psychology and North-West Wales NHS REC approvals (Ref No.07/WNo01/46). Consent for the researcher's right to disseminate photographic content in academic outputs was obtained as part of those approvals.

Following consent, a month before being interviewed on the first occasion, **c**arers were sent disposable cameras and asked to take a minimum of three photographs depicting their typical caregiving experiences. They were advised that their photographs could be of whatever they wished, relevant to their caregiving in either an abstract or symbolic manner, however they were to avoid taking pictures of identifiable persons unless consent was obtained. Caregivers returned brief descriptions of the photographs that they took and an explanation of why they took them (on a provided description sheet).

Once the photographs were developed an interview date was arranged and were conducted in a setting chosen by the participant (Dawn was interviewed at her place of work, Susan was interviewed in her home, Betty was interviewed in a research office within the University). At the outset of the interview the participants chose which of their photos they wished to discuss on each separate occasion i.e., photographs taken prior to the first interview were discussed during each subsequent interview.

The purpose of the photographs was to elicit discussion during these semi-structured interviews; the presence and choice of photographs enabled further insight to be gained into the cognitions and emotions carers held about their experience. Participants were asked about their photograph choices, their caregiving experiences and at the two follow-up interviews they were asked how their experiences had stayed the same or differed since the previous interview. Reflections on the choice of photos over time also allowed caregivers opportunity to reflect on their chronological experience. During follow-ups, carers had ideally to choose between three to five photographs from their original selection.

One participant (Susan) had trouble gaining consent to photograph her mother (recipient) as she had wished to, and she was therefore asked to think of caregiving experiences she most wanted to discuss in conjunction with the two photographs taken and the topics she had raised in the prior interviews.

Adherence to the interview schedule (see Appendix A and B) was not strict and depended upon the degree of participant disclosure. Interviews were audio-taped and subsequently transcribed verbatim.

### Analysis

The second author followed analytical steps recommended by Smith et al. (2009) whereby transcripts were read multiples times making notes of possible themes that emerge for each participant for each time point. Emergent themes were grouped under broader labels enabling contrasts and comparison to be made between each participant and over time. Subsequent cross-case comparisons were conducted. Our procedure maintains the idiographic premise of IPA but also enables convergence and divergence to be assessed between participants and across time. Themes were compared with original transcripts at all stages of the analysis to ensure validity (Smith et al., 2009).

## Results

The nature of returned photographs was diverse depicting physical and caregiving tasks such as cleaning the care recipient's clothes, administering medication or activities the carer or recipient could or could not do. Examples of photos selected by the case studies are presented in Appendix C. For Dawn, her selection reflected her organization of caregiving tasks such as medication dispensing, but also of trying to guide her husband's behavior by signage, and a photograph of the dirty bathroom signified her inability to complete all the chores which she spoke to particularly in the third interview. For Betty the photographs highlighted tasks that she had to frequently complete and which consumed her particularly at the first two interviews, such as cleaning the toilet, but also of aids that helped her physically when she was unable to help (the stair lift) and of means she introduced to her mother so she could keep her belongings tidier (the moveable trolley). Susan did not take many photographs but spoke to issues that reflected frustration at her mother's forgetfulness, including signs she put up around the house in an attempt to correct her mother's inability to remember to shut a door. The photographs were selected by participants at the start of their interviews and offered starting points for conversations and reflections.

**Figure 1:**
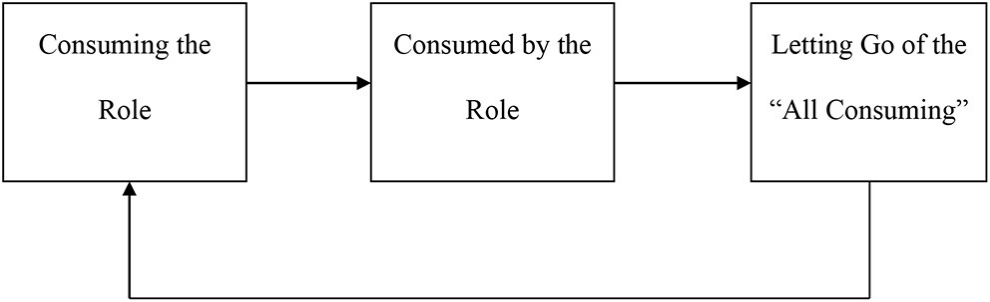
A flow diagram illustration the trajectory between consuming the role, feeling consumed by the role, and letting go of the all consuming. Carers within this study reported feeling torn between these three elements.

Analysis of the interview transcripts revealed three common themes in carer experience. This results section will report on these three themes over time on a case-by-case basis. These themes included *consuming the role, feeling consumed, and letting go*. *Consuming the role* delineates the motivations for caregiving: the different ways and reasons. *Consumed* highlights carers' descriptions of feeling overwhelmed, strained and restricted by caregiving. It describes the causes and impact of feeling consumed over time. *Letting go* describes carers' perception of “letting go” of caregiving either purposively to gain independence or as an eventuality in the future when their recipient deteriorates (see Figure 1). This includes behaviourally or emotionally escaping caregiving.

### Dawn

#### Consuming the Role

During time one interviews all carers found themselves caregiving out of a desire to protect their recipient from harm, and Dawn—whose husband developed early onset FTD—adopted the role as protector because she felt her husband was vulnerable. Simultaneous use of the words “child” and “husband” within the same sentence emphasizes her comparison of the loss of her husband's skills with the need to protect him like a child.

“*You have to watch your child doesn't fall down and graze themselves, you have to watch your husband can use the kettle and not scald himself” (Dawn, 233-235)*

The comparison of her husband with that of a child suggests a sense of role change, which when providing familial care, has been perceived as stressful, for example among female spousal caregivers of husbands with dementia (Vatter et al., 2018) and female spousal carers of stroke patients (Cao et al., 2010). These caregivers reported feeling resentment, anger, sadness and worry for the future as their role transitioned, and as cognitive functioning in their husbands decreased (Vatter et al., 2018). The nature of the relationship was seen as changing, for example caregivers in Vatter's study felt they were providing care not for their husband, but for a more distant uncle, and Cao's wife carers of husbands who had had a stroke felt they had transitioned from “princess to maid,” replacing joint husband-wife activities with provision of instrumental support to their husbands. Engaging in activities which differ from role expectations are termed as non-normative role reversal and taking these on whilst fulfilling usual roles (e.g., being a mother) can result in role conflict, role overload and a sense of burden or stress (Bastawrous et al., 2014). Two of Dawn's photographs highlighted how she took on organizational tasks for example to either prompt her husband's behavior, or to make her own routines around dispensing medications easier.

At the second interview 6 months later Dawn described a desire to consume the role to protect her husband's emotions, to avoid jeopardizing her husband's dignity and as a result she adopted a covert, but organized, approach to providing care.

“*So, I have to de-collect them [her husband hoarded belongings] for him without him noticing... hopefully” (Dawn, 395-396)*

This approach succeeded because it meant Dawn could still be the primary carer without upsetting her husband or causing confrontation. Her desire to protect her husband's emotions resonates with her earlier approach where she had desired to protect her husband from physical harm. This may demonstrate a carer's willingness to adapt their role to meet changing recipient needs in accordance with illness progression. As seen with Dawn, there is evidence that carers adapt to changes even if these are perceived negatively. Carers of those with dementia have reported coping through using denial, control, positive reappraisal, and acceptance and developing an understanding of dementia (van der Lee et al., 2014).

**At the third interview**, although Dawn's husband had deteriorated at an even greater rate than she had expected, it seemed her ability to positively reframe the stressors prevented her from giving up the role. She had initially described the role as an obligation and although the word “duty” is again used at this time point, suggesting she still felt she had a lack of choice, Dawn also described how her belief in her capability and ability to gain satisfaction from caregiving maintained her motivation when faced with adversity.

“*Sense of duty, just wanting to I suppose and knowing I can do it well, and I do get some kind of fulfillment through doing it, where I think it is a bit weird, but I suppose you have to see things positively without going downhill, when you're actually going through it, you turn things into positives if you want” (Dawn, 594-599)*

Dawn focused on the positives in order to adapt to her husband's progressive illness. This is consistent with other evidence, for example carers of stroke or dementia patients (Martin et al., 2006; Williams et al., 2014) and carers of those with spinal cord injury (Dickson et al., 2010).

Dawn's sense of duty to care and her perceived lack of choice accords with previous research. A lack of choice can often be accompanied by carers feeling resentment, although relationship type and quality may also influence this. Lower levels of mutuality in the relationship between carer and recipient resulted in greater resentment; potentially because they missed the life they used to have, whereas conversely carers who had a close mutual carer-recipient relationship reported depression as an outcome as they missed the intimacy of the traditional roles that they used to have (Williamson et al., 1998; Schulz et al., 2012). Resentment and lack of choice are also feelings reported by carers where there is role conflict (Bastawrous et al., 2014). Potentially Dawn felt a sense of duty when considering the change in her role from wife to carer but tried to positively reframe her role change to buffer or prevent any negative outcomes. This did not mean however that she did not sometimes feel that it overtook her.

#### Being Consumed

**At the first interview**, although voicing a desire to care, Dawn's account demonstrated that she also felt constrained and overwhelmed by caregiving and by the multiple demands from other sources such as family and work. Dawn was forced into rationing her time between her daughter and her husband and this role conflict evoked guilt for being unable to fulfill her roles as a mother, wife, and carer, and for giving the false impression that each party had her undivided attention.

“*cos he [husband] does use up so much I do try and give him the impression that he has all that he needs from me” (Dawn, 676-678)*

This follows the scarcity hypothesis with conflict seen between what is expected from the carer and what the carer is able to deliver (i.e., when there is role overload through insufficient time and resources to fulfill the role) (Bastawrous, 2013).

**At the second interview**, Dawn gave an impression of feeling helpless in the face of the sheer volume of tasks and unpredictability in the timing of care demands,

“*I normally quite like things gaugeable but that's, that's less so now” (Dawn, 187-188)*

**By time three**, Dawn's accounts suggested that she was feeling increasingly like a passive agent rather than the active volunteer witnessed in her earlier accounts as her husband's condition continued to deteriorate and his dependency on her grew.

Dawn's perceptions of osing control in the face of growing care tasks can be considered within the illness self-regulation model (IR) (Leventhal et al., 2001) whereby the process of appraising an illness leads to the development of schema consisting in part of perceptions of personal control and treatment control. These combine with perceptions of illness timeline, perceived consequences, illness identity and coherence, emotional representations, perceived causes to influence a person's coping responses and relevant outcomes. Perceptions of personal or treatment control and of illness curability are generally associated with active or problem focussed coping efforts and with positive outcomes, whereas high scores on illness consequences, timeline and identity are typically associated with poorer psychological outcomes (Hagger and Orbell, 2003; Carlisle et al., 2005). Dawn's lack of perceived personal control over her husband's illness progressive timeline could have impacted her sense of well-being and result in poorer psychological outcomes; however some of her future oriented thinking, anticipating role loss, may have buffered this as we see in the next theme.

#### Letting Go

**At the first interview**, some conflict was evident in Dawn's narrative between desiring a degree of freedom from her husband's dependency and her desire to protect him,

“*Not everything has to be about him all the time” (Dawn, 773-774)*

Although Dawn was unable to have physically escaped from caregiving **at the second interview**, she appeared to alleviate her feelings of being overwhelmed by her responsibilities by fantasizing about her life and the freedom she would have in the future, once she was no longer caregiving. This highlights an acceptance that her husband's condition is degenerative and that her caregiving role would inevitably come to an end. Dawn's desire to focus on her own needs in the future emphasizes her current feelings of self-neglect.

“*I quite fancy living a spinster life... not having to think about somebody else and do all the time... there's that constant having to consider somebody else” (Dawn, 604-609)*

Feeling consumed and letting go are interrelated, and it may be that through her wishful thinking Dawn was displaying signs of avoidant or escape coping. Wishful thinking relates to wishing a circumstance was over or was different. Women apparently use wishful thinking more than men and, as a result, are more likely to report higher levels of burden (Papastavrou et al., 2009). Furthermore, research has indicated that wishful thinking increases as a loved one who is suffering from dementia declines cognitively (Gilhooly et al., 2016).

As Dawn felt a lack of control over the growing caregiving tasks (as illustrated in her selecting a photograph of a dirty bathroom) this might have caused her to adopt avoidant coping. A relation between avoidant coping and psychological morbidity has been demonstrated in carers of those with colorectal cancer (Fitzell and Pakenham, 2010), traumatic brain injury (Chronister and Chan, 2006), MS (Pakenham, 2005) and dementia (Kneebone and Martin, 2003) and furthermore amongst carers of people with dementia it has been shown that those using avoidant coping techniques were less willing to continue their caring role (McKee et al., 1999). Carers' wishful thinking and avoidance coping could be considered a mediator of outcomes (Del-Pino-Casado et al., 2011) as suggested in a study of spousal Alzheimer's carers' where escape-avoidant coping mediated the impact of recipients' problem behaviors on the carers' depressive symptoms (Mausbach et al., 2006).

It is well-established that problem-focused coping is more likely when something can be done (or is believed to be possible) to alter the stressor, including illness or its symptoms, while emotion-focussed and avoidant coping techniques are more likely in situations where an individual lacks or perceives a lack of control (e.g., Lazarus, 1993a,b; Lowit and Van Teijlingen, 2005; Schroevers et al., 2007). Relevant here are the findings of an IPA study conducted with dementia family carers where it is suggested carers avoided dealing with the future in order to avoid the anxiety that accompanied being faced with a deteriorating illness (Quinn et al., 2008). Similar avoidant coping behaviors have been reported amongst patients, family members, and family carers who have strong illness identity representations and uncontrollable timeline beliefs (e.g., Hagger and Orbell, 2003; Carlisle et al., 2005).

Dawn seemed to adopt active and information seeking techniques to deal with current problems, but more avoidant techniques when considering the long-term future logistics of the illness and the associated palliative care required. **At the third interview**, in fact, Dawn considered her willingness and ability to care in the future in relation to her husband's deteriorating condition. The fear of her husband's condition further worsening influenced her expectation of being unable to continue with care provision, and it was her perceived lack of physical strength that seemed to be the restriction on her perceived ability.

“*Just the tiredness and inabilities really, I can have some physical limitations like I don't know what I'd do if he was sick or something on the floor cos I wouldn't be able to deal with that so it would only literally be the thing I couldn't manage physically. I can't think of anything that I either emotionally or otherwise might not know how to handle cos I think I'm probably quite strong at everything really so I can't think of anything that would make me give in” (Dawn, 618-626)*

Dawn discussed her future caregiving willingness and motives, where earlier research has asked carers willingness retrospectively. Examining prospective motivations to care we find that Dawn notably was motivated to provide care during the first two interviews while at time three Dawn could no longer envisage herself caring for her husband in the future as his illness continued to progress and demand more physical care. Research suggests difference between willingness and ability or preparedness (Abell, 2001; Parveen et al., 2017) and in wider social cognitive theory (Bandura, 1997) it is recognized that the beliefs in one's ability can affect performance through a distinct but related concept of willingness. In this theory, self-efficacy (SE) is defined as beliefs in one's own ability and capability to succeed. SE varies across different contexts and facilitates perseverance and resilience when faced with adversity. To succeed in meeting one's demands or goals it is necessary to have the skills to execute the task as well as the willingness or motivation to do so. Dawn, who was herself suffering from ill health, felt she did not have the physical ability and skills to carry out the potential future caregiving tasks to the same extent or more intensely than she was at present. Indeed, where carers have self-reported physical impairments, their caregiving role is typically perceived as more overwhelming, leading to increased risk depression and further reduced SE beliefs (Harwood et al., 2000).

Enactive Mastery (EM) is a promotor of SE (Bandura, 1997) and this is the sense of achievement which can be achieved when succeeding in the face of adversity. EM has been shown to mediate the impact of objective and subjective demands of caregiving on negative outcomes such as fatigue, physical strain, depression, and anxiety whilst promoting caregiving satisfaction (Sherwood et al., 2007; Roepke et al., 2009). In times of stress, carers (like Dawn) may draw on a sense of mastery and self-efficacy from past experiences to reduce potential negative consequences of future caregiving demands and this by consequence may increase her willingness to continue the caregiving role in the future.

### Betty

#### Consuming the Role

For Betty—whose recipient was her mother who had previously had a stroke—when she was first **interviewed** her desire to protect her mother was coupled with an unwillingness to relinquish any responsibilities to others because she perceived outside support detrimental to a certain extent.

“*Although it [formal support] was a big help it was also a big hindrance as well” (Betty, 650-651)*

**At the second interview** 6 months later, Betty revealed a determination to care so ingrained that she could not imagine giving up the role.

“*Well it's the natural course of events really, I mean she's cared for me all her life, it just seems natural. There's just no question of anybody else doing it because you know it's, erm... I can't do with people being put in little boxes and put out of the way, it's not right. It just seems natural that after all her caring for me it's my turn to care for her. Just a natural process. I mean when she did go in hospital I was completely lost, completely lost” (Betty, 611-621)*

Use of the term “natural process” provides evidence that Betty thought caregiving for her mum was the right thing to do. Furthermore “natural process” and “it's my turn” additionally suggests that she believed caregiving for her mum reciprocated the care Betty had received when she was a child. This “repayment” motive has appeared elsewhere and can be related to emerging studies of caregiver “choice,” for example, Pertl (2019) who found that perceived choice in adopting the care role amongst 250 spousal dementia caregivers in Ireland was associated with the identification of positive aspects of caregiving.

Betty's determination to continue her role was also based on a fear that formal services would be detrimental to her mother's support. She expressed concern that her mother would be pushed away in a box, implying she believed formal care was impersonal and demoralizing. Her perception of formal services was so negative that she may have even compared her mother's entry into care with that of dying, with the box a likely metaphor for a coffin. Eleven spousal carers to Traumatic Brain Injury patients similarly perceived formal support a hindrance and perceived it prevented carers' gaining a sense of privacy and power, resulting in lack of carers' role adjustment (Dickson et al., 2010). Family carers feel uneasy leaving recipients with strangers in respite and feel it could cause the recipient upset (Montgomery et al., 2002). Contrasting evidence however reveals the positive impact of respite services on caregiving outcomes: reduced caregiver stress and burden; increased quality of life; prolonged longevity of family caregiving in the family or recipient's home; and improved health and well-being of family caregivers and recipients (Sörensen et al., 2002; Gaugler et al., 2005; Etters et al., 2008). The findings from our research, including Betty's experience, suggest a need for emphasis on educating family carers about the benefits of receiving formal support but also for services to tailor support to the individual needs and concerns.

Furthermore, two reasons why family carers do not seek additional support from formal services: not knowing that they can or not perceiving that they need formal support, have been cited (Montgomery et al., 2002). Betty within this study also discusses a lack of need for formal support through beliefs in having an obligation to reciprocate. In a quantitative study of 78 daughters providing support to their elderly mothers, filial obligation or duty was associated with greater perceptions of burden and strain (Cicirelli, 1993). However, when exploring motives using qualitative methodologies, it becomes apparent that carers are able to experience caregiving satisfaction when providing support simultaneously out of obligation and feeling love for their recipient (Hareth et al., 2008; Ribeiro and Paul, 2008). It appears that love and affection are motivators which can coexist with a sense of duty and together these conflicting, seemingly contradictory factors influence the experience of either positive or negative outcomes, but this needs fuller prospective investigation. Our current findings add further insight to Parveen and colleagues' findings (Parveen and Morrison, 2009; Parveen et al., 2011, 2013) that Black and South Asian British carers provided care to their family members out of a sense of obligation whereas White British caregivers provided care due to intrinsic motivations (emotional attachment with the care-recipient). For Betty it appeared that obligation, love and guilt in combination motivated her to continue support without formal care intervention. Additionally, Betty felt determined to care because it gave her a sense of purpose. Her dependence upon providing care is illustrated when considering her reports of feeling a sense of loss when her mother was admitted to hospital after her stroke.

However, at the **third interview**, Betty had relinquished her desire to be the sole carer for her mother and reported having found freedom through employing formal services. Regaining such independence motivated Betty to continue the role.

“*I'm not quite so snappy as I used to be. I used to be “oh I'm fed up of this” you know, “I can't do this anymore” and “I've had enough of that', but no I do it more willingly now. I mean I was willing before, you know, but, it was a drag, but now, I'm sort of happier about what I'm doing... It's made a big difference, big impact” (Betty, 110-115)*

As a result, Betty's attitude changed: she felt destined to care rather than obligated.

“*The fact that we've always been a caring family. My mum's always cared for people such as my nan and my dad when he was ill so it's just a, it's normal to me you know” (Betty, 146-149)*

Perhaps through gaining some distance from the dyadic relationship caregivers can focus on providing tailored and more effective care to the recipient, and this may affect the extent to which carers feel consumed, as discussed below.

#### Consumed

At the first **interview** Betty perceived herself responsible, to a certain degree, for some of the strain she felt at this time. She suggests a loss of control “running about like a mad thing,” attributable perhaps to her determination to take on all aspects of the caregiving role (including aspects perceived to be burdensome and beyond her physical capabilities) without formal support.

“*I'm always so busy that I just haven't got the time and I don't realize until it's bedtime I haven't sat and had a conversation with her you know because I'm running about like a mad thing all the time” (Betty, 30-34)*

“*But I was getting to the stage where I couldn't, I couldn't walk across the room and I certainly couldn't hold a conversation because I hadn't got enough breath” (Betty, 786-789)*

Betty reported resentment was appeared to be confounded by her mother's stubbornness, inability to adapt to the daughter-mother reversed roles, and the demands the mother placed upon her. At this first interview Betty felt consumed because she felt trapped by the role and related tasks (as reflected in her choice of photographs reflecting caregiving demands), one that brought with it a sense of role reversal.

“*It does wear me down at times. Erm, one of the main issues, really, that affects my life is that I'm not allowed out at night. She still thinks I'm six” (Betty, 103-106)*

**By the time of the second interview**, similar to Dawn, Betty's reports suggest she is feeling helpless, with her life being dictated by her mother's illness. Betty jeopardized her own health by placing her mothers' well-being first, and as echoed by Dawn and Susan, the caregiving role took away Betty's autonomy.

“*It's time consuming, that's the problem the time. It just all comes back to time” (Betty, 839-841)*

“*Sometimes if she's shouted me in a panic, I've run upstairs without thinking, I can't breathe when I get there and I can't string a sentence together” (Betty, 721-724)*

The consuming negative impact of caregiving on carer's physical health and mental well-being has been well-documented within caregiving research. **By the third interview** however Betty had gained a renewed quality of life and independence by virtue of accepting and receiving formal support and so at this interview her narrative did not suggest she felt consumed by the role any longer. Her choice of photographs here highlighted aids and adaptations within the house that made things easier for her or for her mother. This highlights the positive impact that some distance from dyadic relationships (through receiving formal support or adaptations) could have on the carer's ability to provide care, including that of “letting go” - our third theme.

#### Letting Go

As said above, at the first **interview** Betty seemed unwilling and reluctant to escape her role, despite feeling overwhelmed by it. Worry for her mother's safety seemed to be what prevented Betty from letting go. Socially isolated, Betty depended on her role to achieve a sense of purpose in life, and she therefore lacked confidence when she envisaged opportunities to gain independence from her mother.

“*I'm going to be left completely on my own and then I'll have to start my life all over again” (Betty, 356-358)*

Ultimately the restrictions her mother placed upon her, almost to the extent of emotional blackmail, left Betty feeling guilty when she did have time away from caregiving. At this first meeting Betty appeared to have given up hope of having any quality of life. Emphasizing her isolation Betty relied on her pet for companionship when there were opportunities to take time out from caregiving.

“*She he's [pet bird] a little bit of distraction for me you know a little bit of pleasure” (Betty, 730-731)*

Betty initially reported feeling guilty for taking time to let go of caregiving and do the things she enjoyed, partly because her mother did not like being left on her own. Similar to Dawn, Betty's accounts here conformed to the “scarcity hypothesis” (Bastawrous, 2013); whereby competing life and caregiving demands resulted in Betty feeling conflicted by what her mother imposed on her and what Betty was able and willing to do. The revised stress and coping model (Folkman, 2008) suggests that the risk of distress would be greater when carers are forced to relinquish perceived personal goals, because there is dissonance between what the carer idealizes and what they are actually capable of achieving.

Betty felt unable to take the time out to socialize, to rest or to commit to attending classes for physical exercise. Anticipating feelings of guilt or a sense of failure through asking for assistance and respite support are common feelings carers experience, and carers report feeling reluctant and guilty when giving up some of their caregiving time or tasks to others because they believe the respite care might be harmful or upsetting for the relative. Anticipatory guilt may cause an individual to reconsider behaving in a certain way and has been associated with increased burden, stress, grief, and resentment (Gonyea et al., 2008; Sanders et al., 2008; Bastawrous, 2013). Care support needs to reassure carers that their sense of guilt is generally misplaced.

**At the second interview** Betty's accounts suggested a shift in thinking–Betty's focus had shifted from improving her mother's quality of life at the first interview to improving her own quality of life 6 months later. This had enabled her to become more assertive in her relationships, illustrated for example by talking to a photograph of a portable trolley she insisted her mother kept her belongings on for ease of access. They had also agreed to give up activities she found demanding such as visiting their caravan.

“*We've left the caravan now. We're not going back. We decided to give it up this year” (Betty, 76-77)*

In fact, **by the third interview** both Betty and her mother recognized the mutual benefits of letting go of aspects of the role and of their future, and almost co-dependent, life. For Betty to maintain the motivation to look after her mother she found she needed to place her own needs and quality of life first. In attending more to her own needs, hobbies and to time away from caregiving tasks, aided by accepting respite care, Betty found that she benefitted from improved mood and mental health and that the relationship with her mother had also subsequently improved. Betty felt empowered from having found some freedom from the role.

“*We've both benefitted. I mean the better I feel, the nicer I am too, where the nicer I am to her, the better she feels, and then, in turn, she is nicer to me” (Betty, 553-556)*

“*So I think she just realized I've got to have a break otherwise I'm gonna crack up and she's gonna have nobody” (Betty, 466-467)*

Taking time out appeared to ameliorate the feelings of being consumed and also of consuming the role. Letting go of some aspects of caregiving acted as a motivator for Betty to continue her role by virtue of its' influence on her emotional well-being, potentially also via gains to physical health.

“*Pressure makes my asthma worse, but when I'm released from my pressure I feel better in myself, and the better I feel in myself, the more I can do, the better I feel, so it's like a vicious circle, it's a nice circle though, instead of being a downward spiral it's an upwards spiral” (Betty, 170-175)*

Although in earlier interviews Betty was seen to have experienced a loss of personal identity and of time for herself, by learning to let go she was better able to experience uplifts, personal growth, empowerment, psychological well-being, a better dyadic relationship with her mum and potentially provided better care to her mum as reported elsewhere (Beach et al., 2000). Although initially experiencing guilt, leaving her mum with respite carers helped Betty experience these positive outcomes. The reported benefits of using respite services, such as decreased burden or strain and increased well-being are not universally reported (Gaugler et al., 2005; Mason et al., 2007; Schoenmakers et al., 2010) although this may be due to methodological problems in many studies including the lack of meaningful control groups and variations in how respite is defined and assessed (Zarit et al., 2017).

At the final interview Betty contemplated her future caregiving role and as seen earlier in Dawn's accounts, at this timepoint Betty also felt unsure that she would be able physically to provide the necessary care should her mother's condition worsen.

“*I just want her to be there as long as possible and that's why I worry a bit about if I can't carry on coping, if she needs medical attention or special care” (Betty, 601-604)*

Betty's experience suggests that service providers should work to enhance carers' acceptance of their need to take time away from the caregiving role and to accept help from others, including formal support services to achieve this, particularly if care needs escalate or carers' own health deteriorates.

### Susan

#### Consuming the Role

**At the first interview**, Susan, who was providing care for her 95-year old mother with dementia, was striving to improve her mother's quality of life, although with different motivation to that suggested by Dawn and Betty's accounts. Susan's caregiving was motivated by a wish to receive appreciation from her mother in return. This “repayment” motive emerges in other discussions, for example in Social Exchange theory (Cook and Rice, 2003; Ejem et al., 2018).

Susan's perception, persisting even at age 64, that her mother preferred her younger brother to herself seemed to encourage her to try harder to win her mother's affection. Although there seemed to be resentment for assisting her mum without her mum showing gratitude “*not a dickie bird'*

“*and I like cooking and I like to cook something that I think she'll enjoy…. And she'll say nothing not a dickie-bird. Not “that was really nice' or “Oh I do like this can we have it again” (Susan, 564-565)*

To our knowledge little research has addressed competition to provide care among adult siblings where a parent requires family care, although several studies have explored perceptions of equivocal caregiving distribution among siblings and rewards from parents for their caregiving (Willyard et al., 2008; Lashewicz and Keating, 2009; Amaro and Miller, 2016). The potential impact of sibling rivalry for attention and affection from their parent (recipient) in a caring context and in terms of the quality of care provided, the carer-recipient relationships, sibling relationships and caregiving outcomes, is worthy of further study.

**At the second interview 6** months later it was her mother's stable condition that had enabled Susan to continue caregiving- her reference to being happy with the “status quo” suggests that her care was conditional.

“*... [when] I'm not busy doing anything I need to be doing, then I'm quite happy with the status quo” (Susan, 2108-2109)*

Susan was happy providing care at this stage whilst her mother's illness did not impinge on her freedom. A nonchalant attitude emitted in the interview gave the impression that Susan was less attached to the role and to her mother than were the other two case study carers in this research. The nature, and the quality of the carer-recipient relationship is important here. A meta-analysis of caregiving studies revealed that adult-child carers are less likely to experience burden as a result of their recipient's physical and behavioral decline than spousal carers (Pinquart and Sörensen, 2003).

Perhaps unexpectedly given the second interview, Susan continued caregiving at the **third interview** despite her mother's deterioration because she did not feel burdened by the role. She attributed the lack of perceived burden to her own attitude, citing her laziness as the reason why she was unmotivated to go to extreme lengths to consume the role.

“*I say nobody else would have done it, erm, and I wouldn't put her in a home, so while it does impact on my life slightly, er, by and large, I mean, I'm really lazy so if I don't have to do something then I think good” (Susan, 831-835)*

Consistent with Bowlby (1997) theory of attachment, the style of attachment that emerged in these interviews appear related to helping behavior. Susan who had a distant relationship with her very old mother was more inclined to detach herself from the role through self-admission of being lazy. Thus, perhaps, insecurely attached, avoidant carers provided less compassionate, emotional and instrumental support (Feeney and Collins, 2001). It could be argued that in contrast to Susan, Dawn and Betty felt more “consumed” by caregiving and that this could be largely attributed to their inability to separate from their loved one. Following from this we might anticipate poorer outcomes for Dawn and Betty, given evidence that carers who consider their relationship with their recipient to be affectionate and valuable are more likely to experience greater depression as the recipient deteriorates and becomes more dissimilar from the person they once knew and loved (Boerner et al., 2004; Hunt and Smith, 2004; Pruchno et al., 2009). Dawn and Betty, who reported a good quality carer-recipient relationship, were more inclined to consume the role to maintain a sense of control and empowerment over the losses of their loved one. However, this resulted in increased carer stress levels, causing detriment to these two carers and potentially their recipients. In effect, consuming the role led carers to feel more consumed by the role. Susan in contrast did not consume the role.

#### Consumed

**At the first interview** Susan stated that her mother's cognitive and behavioral decline were the biggest strains placed upon her life and the photographs of doors where she had placed signs to prompt her mother's behavior were selected to illustrate her frustration with her. Susan reported feeling mentally drained and irritated at her mother's persistent worrying, lack of sensitivity, and forgetfulness.

“*[mother speaking]: “Oh I've been thinking about him [carer's father] all day” [daughter/carer speaking]: and I thought “Oh I could do without this”, you know, “I don't need that” [carer believed her mother was lying and that she had actually forgotten her father's Birthday]” (Susan, 908-909)*

**Six months later** at the second **interview** Susan did not present as any more content with her relationship with her mother and her need to provide care for her.

“*I'm turning into Pavlov's sodding dogs... I'm the one that's being programmed into doing everything and I can't manage to programme her” (Susan, 2074-2080)*

Susan's reference to Pavlov's dogs (conditioned to salivate at the presence of a bell), presents a powerful metaphor. Susan's words convey feelings of being controlled by her mother's dementia; almost as if Susan was the puppet. The comparative metaphor additionally implies she felt emotionally distant, as though caregiving was a mechanical conditioned response, almost to the point of being involuntary. This further supports our theory that a poor pre-morbid carer-recipient relationship influences care provision style. This is further supported when Susan shows that she perceives her caregiving role as more beneficial to her mother than to herself—so much so, that she envisaged herself dying before her mother.

“*Looking at her now, I can see her outliving me” (Susan, 858-859)*

This contrasts with Dawn and Betty who endured their caregiving role through doing more to consume the role as a means of finding meaning, empowerment and positive outcomes from their role.

Dawn and Betty were able to discuss rewards, gains and endurance more than Susan even when they each faced the need to provide instrumental care. This accords with findings that carers in close/mutually dependent relationships who provide instrumental care report greater well-being, consider more of the positives and that their role is worthwhile, endure adversity, and search for meaning and purpose when compared to carers with a distant relationship with their recipient (Ribeiro and Paul, 2008; Poulin et al., 2010; Parveen and Morrison, 2012).

Although previous research has discussed the benefits of a positive relationship with the recipient, it could be argued that an element of maintained independence prevents or buffers carers from experiencing distress. This may help when briefly examining **the third interview** with Susan. While Dawn felt consumed at the final interview, Susan's account suggested that she had been able to resist feeling like she had to do more as her mother deteriorated and by consequence did not report experiencing as much strain as Dawn.

“*I'll do it in a minute and if I don't do it within about ten minutes she keeps coming in; [mother says] “Have you...” but that's it. She remembers but sometimes she's forgotten” (Susan, 1080-1070)*

Perhaps the emotional distance between Susan and her mum provided a means of coping i.e., similar to avoidance.

#### Letting Go

In contrast to Dawn and Betty, at the first **interview** it was clear that Susan found no difficulty letting go, due to the support she received from hired help and the fact her mother was not dependent on her for 24-h care. Susan had been caregiving for her mother for 5 years (in contrast to Dawn and Betty who had provided care for 3 years). Susan's conscience was reassured by formal care support, allowing her to take time away without feeling guilty or worrying about the safety aspects of caregiving and her mother's welfare. It seems Susan's distant relationship with her mother increased her ability to accept help from others.

“*If I didn't have [hired nurse] then I think I'd probably be crawling the wall” (Susan, 419-420)*

Six months later at the second **interview** Susan used care withdrawal techniques with the intention of gaining control when her mother was unappreciative of her efforts. Susan's implied indifference to caregiving facilitated her active refusal to carry out certain tasks and by maintaining an independent lifestyle she was able to prevent herself from feeling trapped. This revealed that Susan could be just as distant from her mother, as she perceived her mother could be from Susan.

“*I don't have to stay here anymore... I can go get a job err go over to [country] where my daughter is... I can actually do anything I want to do” (Susan, 685-691)*

Williamson et al. (1998) described two types of sadness that carer's experience associated with loss: loss of a carer's loved one and loss of the life they used to live, it is possible that Susan maintained distance with her mother to prevent subsequent sadness through loss of her mother, or current sadness at the loss of the life she used to have.

**At the third interview** 12 months after we met Susan, when faced with the prospect of providing more difficult caregiving tasks as her mother's condition worsened—the emotional distance from her mother which had previously worked in her favor when her mother's condition was stable, now became a disadvantage. Susan was left feeling incapable of providing the necessary nursing or instrumental type care.

“*It's sort of like having a baby, it's difficult when it is a close relation, er, because of the mother/child scenario” (Susan, 828-831)*

“*I said to her “look you know I will never put you in a home”. I mean if she got really, really, really, bad and she didn't know that she had to go in a care home. I mean there may come a scenario when she might become violent then I couldn't I don't think I could cope with that... If she became incontinent I would find that quite difficult” (Susan, 818-826)*

Susan's attitude conveyed a lack of affection and motivation and the likelihood she would take on more difficult, personal, and emotional caregiving tasks if her mother's condition deteriorated seemed small. It also reflects findings that where caring tasks are seen as outside or beyond those typical for their role expectations, for example for Susan as an adult-child facing possible personal care needs, then this can create more distress (Savundranayagam and Montgomery, 2010).

Table 1 summarizes the high-level findings within the themes, across case studies and interview time points.

**Table 1:** A table outlining the high-level findings within the themes, across case studies and interview time points.

	**Consuming the role**	**Feeling consumed**	**Letting go**
**Dawn caring for husband with dementia**			
First interview	Yes–to protect recipient	Yes–feeling guilt	No
Second interview	Yes—but a covert approach	Yes—feeling helpless due to volume of care tasks	No–but emotionally escaping and fantasizing about life once no longer caregiving
Third Interview	Yes–by positive reframing the stressors	Yes–feeling less control over the illness trajectory	Yes; more than at the start; felt physically unable to care in the future with recipient and own health worsening
**Betty caring for mother who had a stroke**			
First interview	Yes–lack of trust in formal support services	Yes–felt trapped	No—the role gave the carer a sense of purpose so actively took on the role
Second interview	Determination to care ingrained and a natural process	Yes, feeling helpless due to volume of care tasks	Yes, change of focus-improving her own quality of life—felt assertive to refuse demands placed upon her
Third interview	No—independence of role through accepting formal support	No—renewed QoL through accepting formal support services	Yes, felt physically unable to care
**Susan caring for mother with dementia**			
First interview	Yes, through a desire to be appreciated	Yes–frustration	Yes, through accepting formal support
Second interview	Yes, but attributed to Mother's stable condition.	Yes, felt almost conditioned by mother's dementia related demands	Yes, when her mother was unappreciative of her efforts
Third interview	Yes, but providing the minimal to support to ensure well-being	Yes—feeling ever more consumed by mother's demands	Yes; seen as necessary if feel incapable of providing nursing type care

## Discussion

In this series of longitudinal interviews conducted with three caregivers at three timepoints we sought to gain novel insights into the experiences carers face over time by adopting Interpretative Phenomenological Analysis of interviews enhanced by photograph elicitation.

Carers varied over time between: consuming the role, feeling consumed by the role, and letting go of feeling consumed and whilst themes were interrelated certain themes were dominant in a carers' life at one particular time. This depended on multiple factors such as illness type, which included characteristics of illness timing and predictability of onset and progression/stability, and on carer-recipient relationship type and quality. Initially, Dawn and Betty predominantly reported consuming the role and it having a draining effect on their resources and as a consequence they experienced role conflict, whereas Susan reported the benefits of letting go and letting others help. Betty reported a lack of trust in formal support provision that would have acted as respite and thought it would be detrimental, impersonal and demoralizing. As a consequence she refrained from seeking help from formal support services, and in fact Betty felt the caregiving role gave her a sense of purpose. Over time, of all the themes emergent from the data, “letting go” seemed to provide carers with the most benefit to their quality of life and motivation to continue the role. Indeed, at the later interviews Betty in particular, reported having found freedom, improved quality of life and confidence through accepting a need for home adaptations or formal support for her mother. Previous research has illustrated the benefits of taking time out to focus upon carers' own needs (Betrabet, 2009; Hill et al., 2009), however our data suggests that further education may be needed to reassure family carers of the benefits to both the carer and care receiver, that accepting formal support could offer. Taking time out from their role seems to provide carers with a sense of normality, control and freedom at a time when caregiving may have taken over their life to an extent where they feel entrapped and depressed (Croog et al., 2006; Martin et al., 2006; Levesque et al., 2008).

When anticipating feeling burdened by future caregiving tasks Dawn adopted wishful thinking to escape feeling overwhelmed. Susan and Betty adopted active coping to deal with current tasks as seen in photographs that reflected organization for example, but avoidant coping to cope with future logistics. We saw in the interviews that participants had concerns regarding their ability to deal with future demands or changes as their recipient deteriorated, which we describe as their perceived self-efficacy for anticipated caregiving tasks. For each participant, the expectation of their recipient's illness worsening was coupled with a concern that caregiving would by necessity become more physical or more intense. Self-efficacy appeared to be a major determinant of whether these carers believed they would continue caregiving in the future. Self-efficacy therefore offers a key target for interventions to improve carers' resilience and potentially carers' willingness to care in the future, and importantly this belief was found, in a review of carer focussed, web-based intervention trials, to be amenable to change (Ploeg et al., 2018).

Within this study we found that self-efficacy beliefs varied according to the pre-morbid carer-recipient relationship quality, with the two carers who claimed to have a good prior carer-recipient relationship expecting to feel unable to cope should the “physical” demands increase, whereas the carer who reported a poorer pre-morbid and current carer-recipient relationships expecting to feel unable to maintain her role should the recipient require more “personal” types of care. Such findings highlight a difference in willingness and ability to care, depending upon the type of care required (cf. Abell, 2001; Parveen et al., 2011) and supports previous findings that carer-recipient relationship quality potentially confounds both the extent the carer contributes to providing care and their experience of caregiving (Ribeiro and Paul, 2008; Poulin et al., 2010).

## Strengths and Limitations

A particular strength of this study is its idiographic approach and longitudinal design which we believe has enabled new findings of how carers experience their role over time.

We believe that no previous studies have found that negative caregiving consequences are, in part, under volitional control and yet our data on the underlying reasons for consuming caregiving or allowing themselves to consume, would suggest this may in part be true. This is important because of the implications it holds for interventions. It suggests carers are responsible to a certain extent for ensuring their resilience through balancing their own quality of life and caregiving workload. Services which deliver interventions and support to these carers at risk of doubting their own capability could help to maintain caregiving at home. Interventions which focus on enabling and empowering carers to be resilient, aware of alternative support and comfortable with requesting and receiving support will facilitate them to gain a degree of independence or “letting go” of what has been described within our findings as a consuming role. In this way, carers may experience the role more positively (e.g., Ploeg et al., 2018). Our findings suggest that occasional detachment from the caregiving role works in the carers' (and, it was suggested, in the recipients') favor. Short-term breaks enabled carers, within our study, to feel a sense of freedom and satisfaction in their life, and also enhanced their perceived ability to endure caregiving.

In terms of limitations, the fact that our sample varied in terms of carer and recipient or illness characteristics may have confounded interpretation (Brocki and Wearden, 2006), however the presence of diversity within this study enabled fruitful insights to be gained into a range of caregiving experiences, particularly over time as different conditions changed in different ways. IPA is optimal for analysis of between four to 10 interviews thus the nine interviews we conducted is deemed sufficient for this type of in-depth case-oriented analysis and to meet the stated goals of the study (see also Vasileiou et al., 2018, for a useful review and discussion of sample sizes and their justification within qualitative research).

The study of course also lacks the care recipient perspective concerning the care received and the care-recipient relationship quality. Given that studies of caregiving dyads have shown that perceived relationship quality and its impact differ according to whether the reporting individual is a carer or care recipient (Braun et al., 2009), longitudinal qualitative studies with dyads could perhaps aid understanding of the dynamics of care relationships more fully than is possible here. We are currently conducting such a study. In addition, gender and gendered roles cannot be addressed here as our case studies are all female. Carers UK (2017) report that an increasing percentage of carers are male (44% in the USA, 42% in the UK) and thus previously reported differences in motivators to care based on gender (whereby women experienced greater pressure and obligation to care and less willingness to care than men, e.g., Yee and Schulz, 2000; Pinquart and Sörensen, 2003; Ussher and Sandoval, 2008;) need to be explored contemporaneously.

The mixed-method approach employed in this study, using participant's own selected photographs to structure and aid interview discussions provided insight into carer cognitions, illness perceptions and motivations, related often to specific care tasks or anticipated role expectancies. It could be argued that whilst we advised carers to take photographs they felt represented their situation, thoughts and feelings, they may have felt unable or unsure about taking certain photographs: we as researchers have no control over this and could only work with what was presented. Cognitive interviewing techniques used to assess behavior and perceptions on sensitive topics could perhaps replace the semi-structured interviews employed here to gain further insights into why and how carers differentially perceive and respond to their role over time, in the way that our findings suggest.

## Conclusion

Our unique combination of data elicitation and idiographic analysis succeeded in capturing individual caregiving experiences whilst identifying several common emerging themes that explained carer experience over time. We have shown an interaction between consuming the caregiving role, feeling consumed by that role, and relinquishing the consuming role which varied between participants as a result of situational, relational and personal differences.

Overall, findings from this longitudinal case study highlights the fact that a one-size intervention would not suit all. Our data suggest that some carers feel conflicted between consuming the role and letting go of aspects of it. Feeling consumed appeared to be the price paid for taking on the role when they would have benefitted from service support.

Crucially our data would support that carer individual differences, individual contexts including the quality of the relationship with the care recipient are fully and carefully considered when implementing individual supportive care plans. Basing interventions on group mean data, derived from the many existing quantitative studies of caregiving and its' outcomes, inevitably targets the average person and the average need and has the potential to miss the more idiosyncratic aspects of caregiving and receiving. Enabling carers to meet specific recipient illness-related needs by building their self-efficacy could maintain caregiving motivation, however interventions designed to improve carer well-being need to look beyond meeting care recipient needs in an efficacious manner to consider also the carer's perceptions, motivations and needs. To meet carer needs could simultaneously help reduce the service expenditure arising from the breakdown of informal care networks and the admission of recipients into formal care. Whilst more respite support should certainly be available, we suggest some of this need would be mitigated through more targeted personal support and training for carers.

## Data Availability Statement

The datasets generated for this study are available on request to the corresponding author.

## Ethics Statement

The studies involving human participants were reviewed and approved by Bangor University School of Psychology Ethics Committee; NHS Research Ethics Committee. The patients/participants provided their written informed consent to participate in this study.

## Author Contributions

KW conducted the study as part of her Ph.D. studies at Bangor University under VM supervision. Initial writing was presented by KW with significant updating and restructuring provided by VM. As KW is no longer in academia VM is the designated corresponding author.

## Conflict of Interest

The authors declare that the research was conducted in the absence of any commercial or financial relationships that could be construed as a potential conflict of interest.
