# Supplementary material for: Gaining Longitudinal Accounts of Carers' Experiences Using IPA and Photograph Elicitation
Source: Front Psychol. 2020 Dec 4;11:521382. doi: 10.3389/fpsyg.2020.521382 (PMC7746611; doi:10.3389/fpsyg.2020.521382)
Supplement: Supplementary file 1 [file Data_Sheet_1.pdf]

Appendix A: The First Interview Schedule

Carers pick at most five photographs (minimum three) that mean the most to them. If there are no photographs available ask carers to think of and disclose three to five scenarios to discuss.

Photo-choice/Image-choice

What is it?

Why did you take/why did you imagine it? Prompt: what was happening at the time you chose to take it/of the situation?

Why did you choose to discuss it in this interview?

How does it make you feel?

Any thoughts on why you feel this feeling?

How does this have an effect on any aspect of your life?

How does this affect the care you provide?

What are your thoughts on how this will affect you in the future?

How do you deal with it?

What do you think can be done about it in future?

What do you think you can do about it in the future?

Debriefing

End
