# Supplementary material for: Gaining Longitudinal Accounts of Carers' Experiences Using IPA and Photograph Elicitation
Source: Front Psychol. 2020 Dec 4;11:521382. doi: 10.3389/fpsyg.2020.521382 (PMC7746611; doi:10.3389/fpsyg.2020.521382)
Supplement: Supplementary file 2 [file Data_Sheet_2.pdf]

Appendix B
The Follow-up Interview Schedule

The carer has to choose between three to five photographs they most want to talk about on the day of the interview and are most relevant to how they are feeling at that particular time, on that particular day. For those participants who were not able to take photographs at time one but were willing to describe a situation the participants at time two will again choose between three to five scenarios that are most relevant to how they are feeling on that particular day of the interview and that they most want to discuss. The interviewer will then ask questions relating but not restricted to the following areas:-

Caregiving Background
How long have you been providing care for?
How many hours do you provide care for?
What kind of help/care do you provide?
Photo-choice/Image Choice
What is it? Why did you take it six months ago? Why did you want to talk about it today?
Feelings
How do you feel about it now? Why do you feel like this?
Impact
How does it impact on your life? Why has it impacted on you like this?
How does it impact your motivation to care/how did it impact on your life when you took the photograph/how did it impact on your life at the start? Why has it impacted on you like this?
How does this impact your ability to care? Why has it impacted on you like this?
Expectations
In what ways does what is happening in the photograph meet your expectations of what you thought caregiving would be like?
Coping
How do you deal with what is happening in the photograph?
Future
What do you hope to see happen with this in the future?
How would you like to cope with this in the future?
What is the likelihood of this happening? What will actually happen?
General Willingness and Motivation Questions
What makes you want to care/What makes you not want to care?
How do you see yourself carrying on?
How much do you want to carry on/Why?
How much do you want to stop/Why?
What motivates you to carry on caregiving?
General Choice Photograph/Image Questions
I noticed that the photographs/images you chose to talk about today were: -
The same/…… apart from/were different. I'm really interested to know why this was.
