# Supplementary figures and images for: Gaining Longitudinal Accounts of Carers' Experiences Using IPA and Photograph Elicitation
Source: Front Psychol. 2020 Dec 4;11:521382. doi: 10.3389/fpsyg.2020.521382 (PMC7746611; doi:10.3389/fpsyg.2020.521382)

**Appendix C**  
**Exemplar photographs of case study participants**

Dawn:

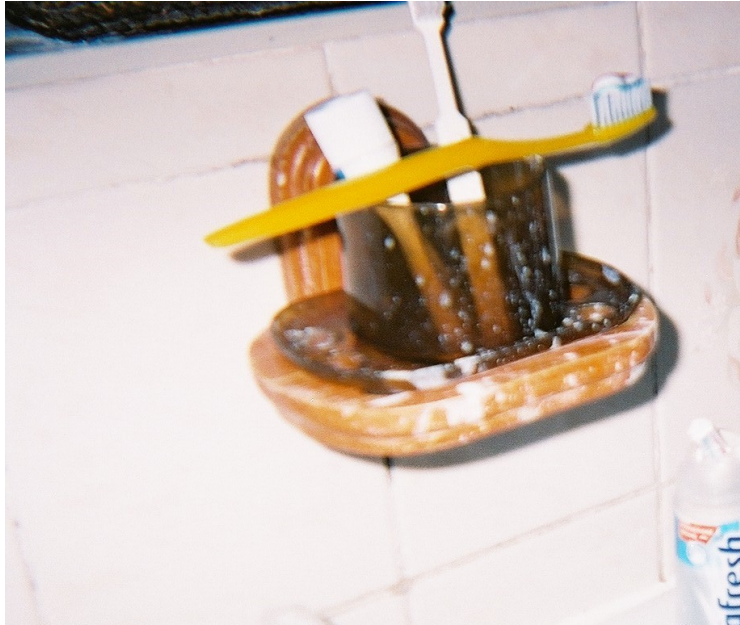

Betty:

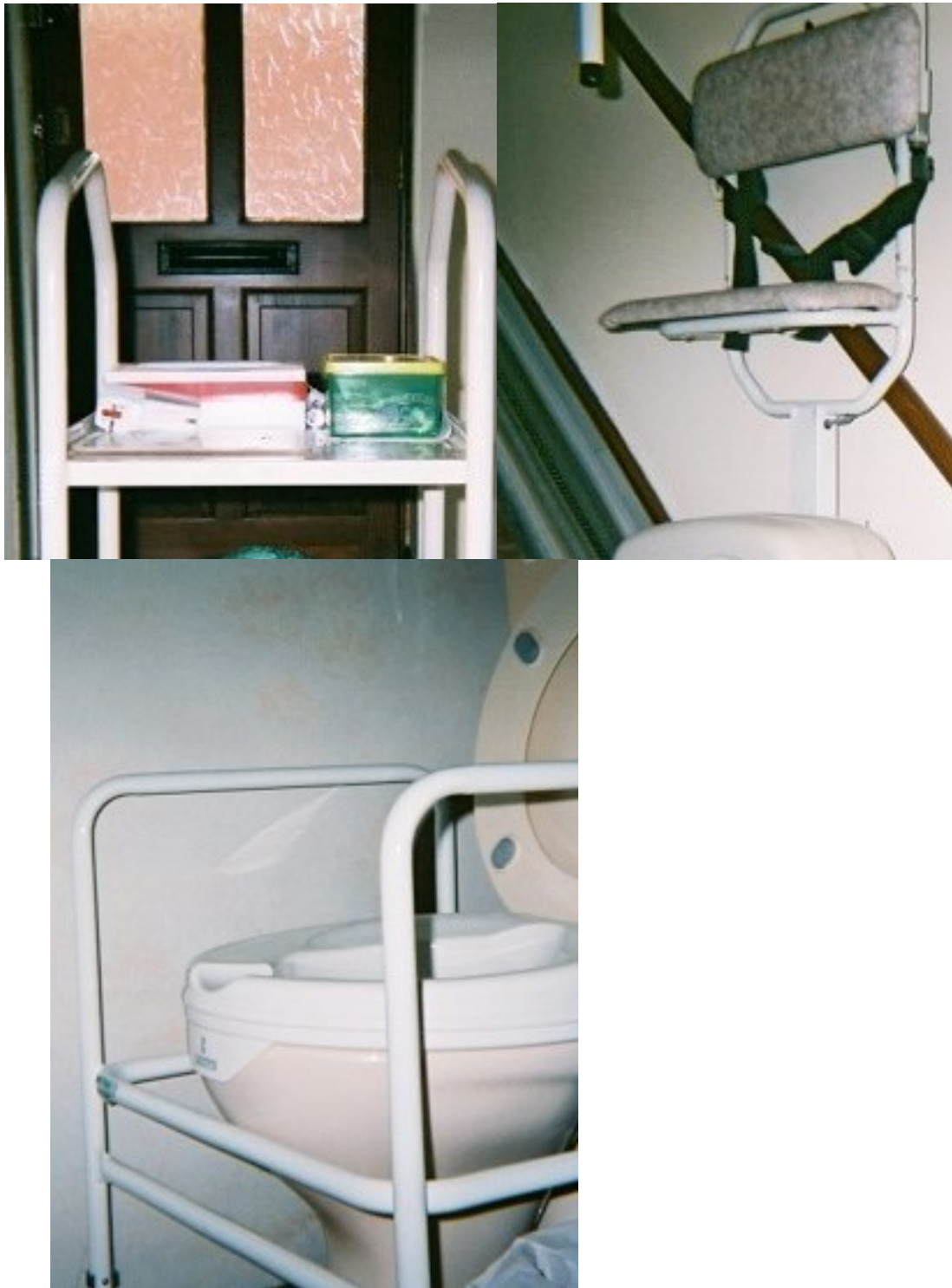

Susan:

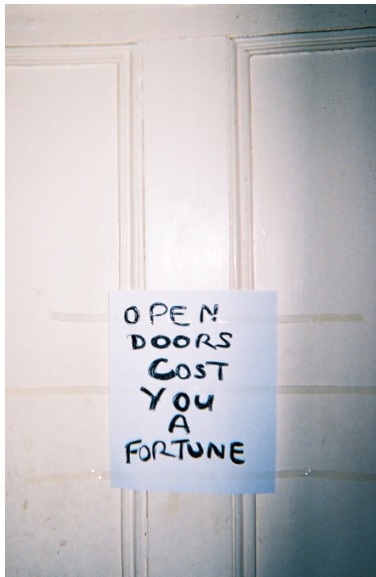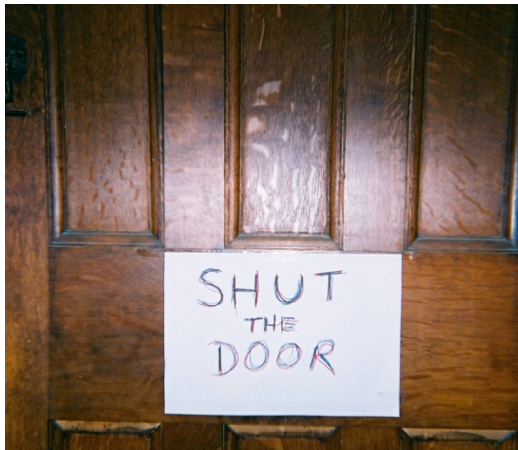

Supplement: Supplementary file 3 [file Data_Sheet_3.pdf]
